# Mental health nurses’ experiences of caring for inpatients with severe mental disorders: a qualitative study

**DOI:** 10.1186/s12888-025-07024-7

**Published:** 2025-07-01

**Authors:** Roghaieh Keykha, Naima Seyedfatemi, Seyedeh Narjes Mousavizadeh, Parvaneh Vasli

**Affiliations:** 1 Department of Psychiatric Nursing and Management, School of Nursing and Midwifery, Shahid Beheshti University of Medical Sciences, Tehran, Iran; 2 Geriatric Mental Health Research Center, Nursing and Midwifery Care Research Center, School of Nursing and Midwifery, Iran University of Medical Sciences, Tehran, Iran; 3 Department of Community Health Nursing, School of Nursing and Midwifery, Shahid Beheshti University of Medical Sciences, Vali Asr Ave., Ayatollah Hashemi Rafsanjani Cross Road, Tehran, Iran

**Keywords:** Nurse, Care, Severe mental disorders, Qualitative research

## Abstract

**Background:**

Patients with severe mental disorders (SMDs) often have complex needs that require long-term, multifaceted care. Exploring the experiences of mental health nurses (MHNs) in this context offers valuable insights into previously underexplored aspects of psychiatric care. The objective of the present study was to examine the experiences of MHNs in providing care for inpatients with SMDs in psychiatric wards in Iran.

**Methods:**

This study used a qualitative research design based on conventional content analysis. The participants were 20 MHNs working in psychiatric wards in Iran, selected through purposive sampling. Data were collected through semi-structured, in-depth individual interviews, with a mean duration of 45 min. The data were then analyzed using conventional content analysis, with the help of MAXQDA (ver.10) software.

**Results:**

The data analysis reduced to the emergence of three main themes and 12 categories, including (1) “care based on the nature of the disease”, comprised of four categories, viz., disease symptoms and specific nature, nursing care to prevent harm to oneself and others, therapeutic communication, and central role of medicinal care; (2) “patient- and family- based care”, with four categories of personalized care, trust building, respecting, understanding, and accepting patients, and family education; and (3) “complex and burdensome care”, containing four categories, i.e., challenges in gender-based care, challenges due to disease symptoms, psychological complications of care, and physical complications of care.

**Conclusion:**

The study results clarified three main themes associated with MHNs’ experiences of caring for inpatients with SMDs. Healthcare managers and policymakers could in this way make use of these findings to improve MHNs’ performance in psychiatric wards and boost up the quality of nursing care for inpatients with such conditions.

**Clinical trial number:**

Not applicable.

**Supplementary Information:**

The online version contains supplementary material available at 10.1186/s12888-025-07024-7.

## Introduction

Severe mental disorders (SMDs) refer to a complex set of syndromes characterized by deficits in a person’s cognition, behavior, and emotion regulation [1]. They also represent disturbances in biological, psychological, or underlying cognitive processes, which ultimately lead to functional problems in one or more activities of daily living [2]. SMDs, such as schizophrenia, bipolar disorder, and major depressive disorder, are highly debilitating and have specific characteristics, such as psychosis with a non-organic cause, disease and treatment duration of over two years, and impaired socio-occupational functioning [3]. It is reported that approximately 1% of the total population worldwide is currently burdened with SMDs [4].

Notably, SMDs have numerous consequences, such as sufferers being more likely to experience physical complications and have a lower life expectancy compared to others in the general population [5]. Moreover, aggressive behaviors and legal troubles [6], high unemployment rates [7], substance use disorders [8], and increased suicide risk [9] have been reported in these patients. It is also worth noting that SMDs are multifaceted and costly, and the hope for their improvement is low [10].

Care is a key element in the treatment and recovery of patients with SMDs [11]. This type of care differs significantly from that for other diseases due to the symptoms and specific nature of SMDs, as well as various factors, such as stigma and discrimination [12]. Providing regular care to sufferers accelerates clinical recovery, reduces relapses and readmissions, improves medication adherence, and lowers the burden of such disorders [13]. The overall goal of care in this context is to help patients achieve community reintegration. Enhancing care quality in this context is associated with better patient outcomes, prevention of readmissions, and a reduction in healthcare costs [14].

Although mental health nurses (MHNs) are capable of preventing some diseases, promoting health, improving overall health in patients with SMDs, and developing integrated physical-mental healthcare initiatives for patients [15], the experiences voiced by patients and nurses reveal a lack of support as well as low nursing performance in terms of psychotherapy. As evidenced in previous research, MHNs involved in inpatient psychiatric care services have thus far failed to meet patients’ expectations, including the expansion of interpersonal relationships. Patients may feel that nurses spend insufficient time with them and may misinterpret some issues [16]. The majority of patients with SMDs are also hospitalized against their will by their families, present the most severe symptoms, and receive lower-quality care. Therefore, there is an urgent need to address these issues to better realize nursing care for such patients and make more efforts to improve it [17].

### Background in Iran

Iran, with a population of approximately 87 million, is composed of 31 provinces, each representing a wide range of socio-economic characteristics [18]. Iran has experienced significant political changes over the past century, including major shifts in its governance structure and changes in the country’s political system. These transformations have had a profound impact on the socio-economic status and mental health of the population [19].

The prevalence of mental disorders in Iran has shown a noticeable upward trend. According to Mirghaed et al. [20], the rate has increased by 31.03% over recent years. Earlier studies also reported a rise from 21% in 1999 to 31.7% in 2015 [21]. By 2019, mental and substance use disorders accounted for 1.9% of the total disability-adjusted life years in the country. Among these, depression, anxiety disorders, and bipolar disorder had the most significant impact on the age-standardized disability-adjusted life years rates. Notably, the overall burden of mental disorders in Iran has risen by 77%, surpassing the global increase of 55% over the same period [19]. This indicates a critical need for comprehensive policy attention and strategic investment in mental health care infrastructure and services.

In Iran, there are approximately 8,600 psychiatric beds and over 123 psychiatric care centers offering specialized care to patients with chronic mental illness. Psychiatric nurses, assistant nurses, and service providers are the primary personnel working in such centers. Nurses are typically required to hold a bachelor’s degree or higher in nursing in order to practice. Assistant nurses generally complete a one-year education program in the field of nursing and perform routine patient care under the supervision of nurses. Service providers undergo a six-month education program in psychiatric wards and are responsible for performing basic patient care activities, such as activities of daily living [22].

The Bachelor of Science in nursing program in Iran includes both theoretical and clinical training in psychiatric nursing. The theoretical curriculum consists of 68 h, after which students participate in clinical placements in psychiatric settings to apply their knowledge and skills. These placements, conducted in psychiatric wards, typically involve two 9-day training periods supervised by nurse instructors [23].

Graduates with a bachelor’s degree in nursing often begin working in psychiatric wards without specialized training. As a result, many psychiatric nurses lack the knowledge and skills necessary to effectively care for individuals with mental illnesses. Moreover, the selection process for these roles often fails to identify suitable candidates. Notably, some nurses are reassigned to psychiatric wards due to poor performance in other nursing departments [22]. Currently, a limited number of Iranian medical universities provide specialized training in psychiatric nursing, but this is insufficient to meet the nationwide demand for psychiatric staff. Additionally, some psychiatric nursing graduates pursue a PhD in nursing and transition to academic roles as faculty members.

### Review of literature

Much research has been conducted to date to explain MHNs’ experiences of caring for patients with SMDs. According to a qualitative descriptive study, Holmberg et al. [24] described the experiences of registered nurses in assessing patients with SMDs during emergency care, with the main theme labeled “conditional patient assessment”, which included two categories: “challenged professional role” and “limited openness for patients”. In another qualitative study of experiences shared by patients and medical staff regarding the therapeutic needs of hospitalized patients with psychosis in London, England, Wood et al. [25] identified themes such as “facilitators of care”, “treatment complexity”, and “a combination of social factors and quality care delivery limitations”. Similarly, Lundström et al. [15] examined MHNs’ experiences related to physical healthcare services and health promotion for patients with SMDs, and identified three themes: “focus on health promotion in each encounter”, “support for unique mental prerequisites of each patient”, and “responsibility for health promotion at all organizational levels”.

As shown in a qualitative study in the Netherlands, Voort et al. [26] reflected on MHNs’ perceptions of their role in physical screening and lifestyle coaching for patients with SMDs, where most participants declared such interventions as an integral part of their professional performance. Nevertheless, they acknowledged a discrepancy between their perceived and actual performance and referred to building therapeutic relationships as a significant but demanding part of improving physical health conditions in patients. In a qualitative study conducted in Iran, Zaree et al. [22] investigated the challenges encountered by nurses working in acute psychiatric departments. The findings revealed that nurses experienced four distinct categories: “experiencing psycho-social challenges,” “experiencing psychological challenges,” “encountering catalysts causing challenges,” and “employing various strategies for coping with challenges.”

Most of the existing research on the experiences of MHNs has focused on patients with acute conditions and physical care needs, while less attention has been given to long-term care for patients with SMDs in specific cultural contexts, such as Iran. This study aims to fill this gap by exploring the experiences of MHNs in Iran, with a focus on the unique cultural and structural challenges of the Iranian mental health system.

As such, experiences vary in all individuals with different nationalities and cultural backgrounds and could be described in unique ways. Therefore, conducting more studies in this field across the world is of importance [27]. In this regard, qualitative research could provide an opportunity for in-depth investigations, perceptions, and comprehensive identification of this phenomenon [28]. Exploring MHNs’ descriptions of the Iranian context and culture could thus be effective in increasing care quality, expanding health outcomes in patients with SMDs, and resulting in family satisfaction. Against this background, the present study aims to explain MHNs’ experiences of caring for inpatients with SMDs in psychiatric wards in Iran.

## Methods

### Study design and setting

This study employed a qualitative research design, utilizing conventional content analysis as the primary analytical method. Content analysis enables researchers to systematically and objectively describe research phenomena at the theoretical level. It is versatile, applicable to a variety of document types, including interview transcripts, speeches, and visual materials. Through this approach, researchers can develop concepts, categories, and themes, which may be further expanded to construct models, conceptual frameworks, and maps that comprehensively describe the subject under investigation [29]. The study adhered to the Consolidated Criteria for Reporting Qualitative Research (COREQ) checklist to ensure rigorous and transparent reporting of the research findings [30].

The study was conducted across three hospitals in Iran. Talaqani Hospital, affiliated with Shahid Beheshti University of Medical Sciences in Tehran, houses a psychiatric ward with 12 beds each for male and female patients. The wards are separated by a metal partition and are staffed by 12 nurses and two nurse aides. Imam Hussain Hospital, a general hospital also affiliated with Shahid Beheshti University of Medical Sciences, Tehran, includes four psychiatric wards: a children’s ward (20 beds), a women’s ward (30 beds), a men’s ward (30 beds), and an emergency ward (10 beds). Participants for this study were selected from the women’s and men’s psychiatric wards, each staffed by 10 nurses and one nurse aide. Baharan Hospital, a specialized referral psychiatric hospital affiliated with Zahedan University of Medical Sciences, is located in southeastern Iran. It consists of three male psychiatric wards with a total of 64 beds, employing 31 nurses (both male and female) and four male nurse aides. Additionally, the hospital includes a 30-bed female ward, staffed by 12 nurses and one nurse assistant, as well as a 27-bed emergency department with 13 nurses and two nurse assistants.

Patients diagnosed with schizophrenia, bipolar disorder, and major depressive disorder—collectively referred to as patients with SMDs—are admitted to either the women’s or the men’s ward according to their gender. None of the three hospitals have dedicated rooms specifically for patients with SMDs. In instances where patients display uncontrollable aggression, they may be temporarily transferred to an isolation room to ensure the safety of both the patients and others. All nurses employed in these hospitals possess a bachelor’s degree in nursing.

### Participants

The study participants consisted of MHNs working in psychiatric wards. A total of 20 qualified nurses were recruited through a purposive sampling technique. The inclusion criteria required participants to have at least six months of work experience in psychiatric wards and to hold a minimum of a Bachelor’s degree in nursing. Additionally, participants were required to have direct experience in caring for patients with SMDs and demonstrate a willingness to participate in the study. To ensure maximum variation and obtain rich data, efforts were made to include nurses of different age groups, genders, nursing positions, and educational backgrounds (including both Bachelor’s and Master’s degrees). In total, 20 participants were included in the study, comprising 18 MHNs and 2 head nurses from psychiatric wards.

Sampling continued until theoretical saturation was achieved, signifying that no new data emerged. Saturation was considered to have been reached when the interviews ceased to provide novel ideas, categories, or overarching themes. This conclusion was drawn through concurrent data collection and analysis, in which each interview was coded and reviewed before proceeding to the next. The decision was supported by the observation that the final two interviews reiterated previously identified information, offering no new perspectives or conceptual diversity. Additionally, no new patterns or meanings emerged, and further data collection was deemed unlikely to enhance the existing themes. Based on these consistent indicators, the research team concluded that saturation had been satisfactorily reached.

A total of twenty participants were selected for this study from the psychiatric departments of three hospitals: four participants from Talaqani Hospital, six participants from Imam Hussain Hospital, and ten participants from Baharan Hospital.

### Data collection

Data collection was conducted by the first author, a PhD candidate in nursing with a Master’s degree in psychiatric nursing and formal training in qualitative research methods. Semi-structured, in-depth individual interviews were carried out between December 2022 and February 2023. Prior to interviews, informed consent was obtained, and participants completed a demographic questionnaire detailing their age, gender, education, professional experience, and occupational role. Interviews were scheduled during participants’ work shifts, taking place in private interview rooms or educational classrooms within the psychiatric wards, based on participants’ preferences. The interviewer endeavored to establish rapport and create a safe, supportive environment to encourage open sharing of experiences.

Each interview began with open-ended questions (Suppl. 1), followed by exploratory probes to elicit deeper insights. Throughout the process, efforts were made to verify the completeness and accuracy of responses by paraphrasing or summarizing key points for participant confirmation. As data collection progressed, interviews became progressively more focused, leading to the refinement and enrichment of emerging categories.

Interviews lasted between 30 and 60 min (average 45 min) and were audio-recorded with participant consent. Data saturation was achieved when no new themes or perspectives emerged. All interviews were transcribed verbatim and repeatedly reviewed to ensure accuracy and facilitate a comprehensive analysis.

### Data analysis

Data analysis was conducted using the conventional content analysis approach, a widely utilized method in studies aimed at systematically examining text properties and their associated phenomena. This approach facilitates an integrated understanding of the text and its context, enabling the researcher to interpret social phenomena both subjectively and scientifically [31]. For this study, the steps outlined by Granheim and Lundman [32] were followed.

Upon completion of each interview, the recorded content was reviewed multiple times and transcribed verbatim using Microsoft Word 13. The transcriptions were then read thoroughly to establish a preliminary understanding of the content. Following this, semantic units were identified, and the first researcher assigned codes to these units. The codes were compared for similarities and differences, grouped into categories, and further synthesized into overarching themes. To ensure consistency and reliability, the research team engaged in discussions to review the extracted codes, categories, and main themes. Data collection and analysis were conducted simultaneously and continued until data saturation was achieved, at which point no new codes emerged, and categories were repetitive [33]. The data management and organization were supported by MAXQDA software (version 10).

### Trustworthiness and authenticity

To ensure the validity and reliability of the data, the four general criteria developed by Lincoln and Guba [34] were applied. Specifically, credibility was achieved through extended engagement with the participants and their data. This included allocating sufficient time for data collection, establishing trust via effective communication, and conducting member checking by presenting the transcribed interviews to six participants, which facilitated feedback and refinement of the findings. Additionally, triangulation was employed by comparing and contrasting the data with other sources, including external observations from two experts in qualitative research, to further strengthen credibility.

Dependability was ensured by assessing the consistency between the statements, codes, and emerging categories, with validation by both the research team and two external reviewers, who provided an additional layer of reliability. Confirmability was enhanced by presenting direct quotes from participants after extracting them from the interviews, ensuring transparency and objectivity in the data analysis. Finally, transferability was promoted by providing a detailed description of the research process, participants’ characteristics, and the study setting, thereby facilitating the potential application of the findings to similar contexts.

## Results

The mean age of the study participants was 33.75, and their mean work experience in psychiatric wards was 6.05 years. Additionally, 25% and 75% of the participants were male and female, respectively. Other demographic characteristics are provided in Table 1.

**Table 1:** Demographic characteristics of participants

No	Age	Gender	Education	Marital status	General work experience	Psychiatric wards work experience	Type of shifts	Position	Type of employment
1	25	Female	BS	Single	3	3	Rotating	Nurse	Two-year contract
2	35	Female	MS	Married	10	6	Rotating	Nurse	Permanent
3	28	Male	BS	Single	5	2	Rotating	Nurse	Contractual
4	38	Female	BS	Married	12	10	Rotating	Nurse	Permanent
5	24	Female	BS	Single	2	2	Rotating	Nurse	Two-year contract
6	32	Male	MS	Single	6	2	Morning	Head nurse	Permanent
7	31	Female	MS	Single	4.5	2	Rotating	Nurse	Two-year contract
8	28	Female	BS	Married	6	5	Rotating	Nurse	Temporary-to permanent
9	32	Female	BS	Married	9	9	Rotating	Nurse	Temporary-to permanent
10	35	Female	BS	Married	12	6	Rotating	Nurse	Temporary-to permanent
11	34	Male	BS	Married	7	4	Rotating	Nurse	Temporary-to permanent
12	32	Female	MS student	Single	9	6	Rotating	Nurse	Temporary-to permanent
13	29	Female	BS	Married	4	4	Rotating	Nurse	Two-year contract
14	44	Female	BS	Married	20	2	Morning	Nurse	Permanent
15	44	Female	BS	Married	20	16	Morning	Head nurse	Permanent
16	28	Male	BS	Single	5	5	Rotating	Nurse	Temporary-to permanent
17	31	Female	BS	Single	7	6	Rotating	Nurse	Permanent
18	48	Female	BS	Married	18	10	Morning	Nurse	Permanent
19	36	Female	BS	Married	6	2	Rotating	Nurse	Temporary-to permanent
20	47	Male	BS	Married	23	14	Morning	Nurse	Permanent

During the data analysis, three main themes emerged, including “care based on the nature of the disease,” “patient- and family-based care,” and “complex and burdensome care” (Table 2), along with the relevant categories, which are explained below.

**Table 2:** Themes and categories extracted from the study data

Themes	Categories
Care based on the nature of the disease	− Disease symptoms and specific nature − Nursing care to prevent harm to oneself and others − Therapeutic communication − Central role of medicinal care
Patient- and family- based care	− Personalized care − Trust building − Respecting, understanding, and accepting patients − Family education
Complex and burdensome care	− Challenges in gender-based care − Challenges due to disease symptoms − Psychological complications of care − Physical complications of care

### Care based on the nature of the disease

As highlighted by the participants, the care provided to inpatients with SMDs in psychiatric wards was largely influenced by the nature of the disease. The main theme, “care based on the nature of the disease,” emphasized that care should be tailored to the patients’ symptoms and conditions. This theme encompassed four distinct categories: “disease symptoms and specific nature,” “nursing care to prevent harm to oneself and others,” “therapeutic communication,” and “the central role of medicinal care.”

#### Disease symptoms and specific nature

One of the categories under “care based on the nature of the disease” identified in this study was “disease symptoms and specific nature.” According to the participants, inpatients with SMDs in psychiatric wards exhibited a range of symptoms that significantly impacted the delivery of healthcare services. These symptoms and the specific nature of the disease included agitation, irritability, delirium, visual impairments, and frequent relapses. The participants emphasized that managing these symptoms, particularly within the caregiving context, posed significant challenges. For instance, a head nurse with 16 years of experience in psychiatric wards shared the following observation regarding delusions:

*One thing that really stands out in patients with severe mental disorders is their delusions*,* and I think it’s something we really have to consider while caring for them. Like right now*,* there’s this patient in our ward who keeps thinking his wife cheated on him. It totally affects how he acts and talks—he’s always lost in his own world. So during care*,* we’re constantly trying to keep him from sinking deeper into those thoughts.*(Participant14)

#### Nursing care to prevent harm to oneself and others

“Nursing care to prevent harm to oneself and others” was another category representing the measures taken by MHNs in caring for inpatients with SMDs and preventing them from harming themselves or others during their stay in psychiatric wards. Such care services included coping with suicidal thoughts, managing aggressive patients through environmental, communicative, physical, and chemical interventions, as well as efforts to maintain the physical safety of both nurses and patients. Despite the inherent challenges of this type of care, most nurses viewed it as a necessary and unavoidable part of their professional role and made efforts to perform it as effectively as possible. Regarding nursing care for patients with suicidal thoughts, one of the study participants illustrated their experience in this way:

*If a patient has suicidal thoughts*,* we do check-ups at random times*,* not at a fixed hour. We also make sure to remove any sharp or dangerous stuff around them. After that*,* we try to keep the patient in sight*,* and always make sure there’s a small light with dim lighting in their room*,* so they’re easier to see. Another thing we do is not place these patients in the last rooms. When handing over the shift*,* we make sure to let the other nurses know by assigning a special code. In our hospital*,* it’s code 12*,* and we write it on the board so everyone knows the patient is suicidal and needs extra attention*,* especially in the middle of their stay*,* because that’s when the risk is higher*,* as they start getting more energy and strength*. (Participant13)

With respect to the management of the patient’s aggressive behaviors, another participant, with 18 years of experience as a nurse in a permanent position, mentioned that:

*When you’re dealing with an aggressive patient*,* the first thing that really matters is figuring out what’s causing it. You’ve gotta talk to them*,* watch their behavior*,* try to get where it’s coming from. Sometimes it’s just a simple request you can actually meet*,* and other times it’s because of delusions—but either way*,* we have to explain things to them.* (Participant 18)

#### Therapeutic communication

The study participants shared their experiences of caring for inpatients with SMDs based on their specific nature, particularly in the context of therapeutic communication. This included providing effective communication conditions, evaluating communication achievements, and, in some cases, neglecting communication therapy despite its importance. Nurses recognized the significance of therapeutic communication in managing patients with SMDs, but practical challenges, such as heavy administrative workloads and time constraints, sometimes led to neglecting this aspect of care. Participant 6, a male head nurse with a master’s degree, shared his experience regarding the importance of communication skills in managing challenging situations with patients:

*Being able to communicate well as a nurse is super important. These patients usually get really agitated as soon as they realize they’re being hospitalized*,* they get aggressive and start swearing*,* and they won’t even wear the hospital clothes ‘cause they don’t accept that they’re sick. So*,* in these situations*,* the nurse has to use both communication and technical skills to calm them down and get them to cooperate*,* no matter what.*

#### Central role of medicinal care

“Central role of medicinal care” was one of the categories of “care based on the nature of the disease,” which denoted devoting significant attention to pharmaceutical care in inpatients with SMDs as a prerequisite for their recovery. As stated by the participants, such patients could refuse to take medications due to lack of insight or be under the influence of disease symptoms, such as delusions and hallucinations, or merely collect medications with suicidal thoughts. Certain pharmaceutical care services could therefore be delivered by MHNs through timing the right times to take medications, having patience, prioritizing pharmaceutical treatment, observing patients, and encouraging their cooperation.

However, nurses described this as one of the more challenging aspects of care, often involving patients who were uncooperative or mistrustful. Despite the emotional burden, most nurses viewed this responsibility as essential and approached it with patience and commitment. Referring to having patience during pharmaceutical care, one of the participants, a nurse with 16 years of experience in psychiatric wards, stated that:

*A nurse needs to really know what meds their patient is on—especially psych meds*,* ‘cause they come with a bunch of side effects. And the nurse is usually the first one to catch them*,* since they’re the ones spending the most time with the patient. Like*,* if the patient’s taking lithium*,* the nurse’s gotta know how it works*,* what kind of care it needs*,* how the patient should take it*,* and what serious side effects to watch out for—so they can step in before anything bad happens.* (Participant 15)

### Patient- and family- based care

The second theme “patient- and family-based care” represented the participants’ experiences of much focus on inpatients with SMDs and their family during care delivery. It comprised of four categories, namely, “personalized care”, “trust building”, “respecting, understanding, and accepting patients”, and “family education”, as described below.

#### Personalized care

“Personalized care”, as a category under “patient- and family-based care,” implied dedicating much attention to inpatients with SMDs and the specific conditions in care plans. This was clarified by organizing care with regard to patient’s clinical conditions, bolstering sense of worth in patients, giving patients good opportunities to express their feelings and emotions, and overlooking social and clinical differences of patients during care. Although all participants acknowledged the importance of personalized care, their perspectives reflected different aspects of this concept, shaped by their individual experiences, professional roles, and clinical contexts. One participant highlighted the importance of tailoring care despite similar

*Just because two patients have the same diagnosis doesn’t mean they need the same kind of care. You might have two people with schizophrenia*,* but their symptoms can be totally different. So the way we take care of them has to be tailored to each person—it’s not one-size-fits-all.* (Participant 9)

#### Trust building

One of the categories of “patient- and family- based care” was “trust building”, as the basis of providing care for inpatients with SMDs, according to the study participants. MHNs could thus adjust and implement care by giving more importance to building trust and reflecting on the methods to boost them in patients and their families. Trust building was seen not only as a foundation to begin care but also as a key factor in facilitating emotional expression, enhancing patient cooperation, and ultimately improving care quality. As an example, one of the participants, a young nurse with 3 years of experience, shared one’s experience about trust building in inpatients with SMDs like this:

*I always do my best to make the patients feel safe and trust me. Most of the time*,* they come with their families*,* so I either don’t let the family in*,* or I just go along with what the patient says*,* so they don’t feel like we’re all working together to upset them.* (Participant 1)

#### Respecting, understanding, and accepting patients

Another category under “patient- and family- based care” was “respecting, understanding, and accepting patients”, which included understanding and accepting patients’ feelings and thoughts, paying respect to patients, and reaching positive outcomes. This approach was generally described by the nurses as a constructive and meaningful experience that contributed to therapeutic relationships. In this line, nurse with 10 years of experience in psychiatric wards commented regarding understanding and accepting patients’ feelings and thoughts, as follows:

*With these kinds of patients*,* you’ve got to know that it’s their thoughts and emotions that get all messed up. For me as a nurse*,* it really matters to understand that this is part of the illness. So if they say or do something weird or out of the ordinary*,* I try to get where it’s coming from and just focus on taking care of them.* (Participant 3)

#### Family education

“Family education” was another category of “patient- and family- based care. Nurses expressed that family education was considered an important and beneficial aspect of the care process, contributing positively to the overall care. They emphasized that educating families at the right time with appropriate content regarding the disease and patient care was essential for providing high-quality care. One of the participants said that:

*I don’t teach at a specific time. Every time family caregivers come*,* and I notice that the main caregiver is calm and doesn’t seem stressed or anxious*,* I try to teach them some important things.* (Participant 8)

### Complex and burdensome care

According to the participants’ experiences, the third theme related to caring for inpatients with SMDs in psychiatric wards was “complex and burdensome care”, referring to the adversities caused by providing care for these patients experienced by MHNs. Notably, “complex and burdensome care” consisted of four categories, including “challenges in gender-based care”, “challenges due to disease symptoms”, “psychological complications of care”, and “physical complications of care”, as elucidated below.

#### Challenges in gender-based care

The first category of “complex and burdensome care” was the challenges of caregiving based on the patient’s gender, describing the difficulty of care due to patient’s gender and represented a series of hardships facing the participants to care for inpatients with SMDs, whether male or female. For instance, one of the participants with a Master’s degree in psychiatry asserted that:

*Taking care of female patients is way more challenging because they really miss their kids and husbands. They were used to cooking and cleaning*,* but once they don’t have anything to do*,* their worries and thoughts just multiply. They always ask us to call their families*,* sometimes leave the ward*,* or ask us every minute when their family is coming to see them. It really makes it hard to take care of them.* (Participant 7)

Another participant also talked about the difficulties of caring for male inpatients with SMDs, as follows:

*High physical strength is one of the things that makes taking care of men more difficult. All patients can get involved*,* but when a fight breaks out between the men*,* it gets really intense. They end up beating each other*,* and one of them usually gets seriously hurt by the end of the day. Separating them and managing that kind of situation is really tough.* (Participant 16)

#### Challenges due to disease symptoms

Most of the participating nurses described the care of patients with severe psychiatric symptoms as extremely challenging and exhausting. The reasons cited included the patients’ detachment from reality, bizarre delusions, unpredictability and uncontrollability of symptoms, limited interaction, severe behavioral manifestations, resistance to medication, lack of insight, concealment of symptoms, and poor responsiveness to education. One participant highlighted it like this:

*Caring for all patients can be challenging*,* but it’s way harder for patients with severe mental disorders because they have their own set of symptoms. For example*,* they tend to be very skeptical and don’t always cooperate with nurses when it comes to taking their medications or even eating.* (Participant 19)

#### Psychological complications of care

The third category of “complex and burdensome care” was “psychological complications of care”. Most MHNs had accordingly experienced psychological complications over time after taking care of inpatients with SMDs. Accordingly, these unpleasant experiences included being embarrassed by working with patients, feeling afraid of patients with SMDs, having stress induced by care, being unresponsive to care, feeling annoyed due to care, being irritable under psychological pressure, and having a sense of uselessness. With regard to feeling afraid of inpatients with SMDs, one of the participants, a male nurse with a Bachelor’s degree, affirmed:

*Honestly*,* I’m scared of these patients. Especially during night shifts*,* I always have this fear that they might get aggressive and come at me*,* ‘cause we’re alone. And ever since I heard that something like that actually happened to one of my coworkers*,* my fear’s gotten worse.*(Participant 4).

#### Physical complications of care

“Physical complications of care” were the last category related to “complex and burdensome care”. According to the study participants, they had experienced physical complications after years of caring for inpatients with SMDs. Some negative physical consequences caused by physical restraints, physical problems due to care, and fatigue following multiple tasks were thus included in this category. One of the participating nurses, who was young and had 2 years of work experience, talked about the physical complications of care in this way:

*My lungs are messed up now—like I’ve been a smoker myself. I caught COVID once*,* and when I saw my chest X-ray*,* I honestly felt embarrassed. The problem is*,* there’s no smoking area in this ward*,* so most of the patients just smoke right here. We’re constantly breathing it in*,* always like secondhand smokers. It’s obvious our lungs are full of smoke. But I just deal with it*,* ‘cause honestly*,* these poor patients really love their cigarettes.* (Participant 5)

## Discussion

The study results demonstrated that the Iranian MHNs experienced caring for inpatients with SMDs under three main themes, viz., “care based on the nature of the disease”, “patient- and family- based care”, and “complex and burdensome care”.

The first theme, “care based on the nature of the disease,” emphasizes the need for a comprehensive approach in caring for patients with SMDs. This includes careful monitoring of symptoms, preventing harm to the patient and others, utilizing therapeutic communication to enhance cooperation, and managing medications to avoid complications. MHNs must balance all these elements to ensure effective and safe care for patients. Consistent with this finding, Zarea et al. [35], in a phenomenological study conducted in Iran, extracted “being engaged with patients” as a main theme, including the categories of struggle for monitoring and control, safety concerns, and support for physiological and emotional needs, which aligns with the present study’s emphasis on symptom control, patient safety, and emotional support. However, Daliri et al. [36], in their study conducted in Ghana, highlighted individual factors such as the patient’s condition at presentation, medication side effects, and poor adherence and insight. Although these factors similarly emphasize the importance of pharmaceutical care and patient cooperation, they showed some differences due to contextual variations and healthcare system differences, including disparities in healthcare infrastructure, cultural attitudes toward mental illness, and access to resources. Therefore, although there are overall similarities across different settings, the extent of focus on certain aspects of care, such as challenges in medication adherence, may vary depending on cultural and structural contexts.

Another aspect of “care based on the nature of the disease” involved nursing care aimed at preventing harm to oneself and others by managing environmental, communication, physical, and medical factors triggering aggression in patients. Consistent with these findings, a qualitative study by Välimäki et al. [37] found that positive attitudes, effective communication, structural changes, and self-management skills in nurses and patients could prevent aggression. Comparing these results with the present study, both highlight the importance of communication and environment, but the current study emphasizes personalized care and the role of nurses’ skills in managing aggression. Similarly, Sim et al. [38] also addressed managing patient aggression and suggested strategies for controlling it, aligning with the present study’s focus on trust-building and family involvement. While previous studies focus on communication and environment, this study uniquely emphasizes the holistic approach, combining personalized care with family-centered support in managing aggression.

In view of that, the first theme of the present study about “care based on the nature of the disease” revealed that MHNs needed to gain sufficient knowledge about the nature of SMDs and even clinical competence in disease management and patient care in order to improve the health of patients and themselves, and finally protect other patients from aggressive behaviors in psychiatric wards.

The second main theme related to caring for inpatients with SMDs in psychiatric wards was “patient- and family-based care”, emphasizing the importance of a personalized approach that prioritizes the unique needs and conditions of each patient. Key elements of this care included building trust between nurses and patients, respecting, understanding, and accepting individual differences, and educating families to better support the patient. Consistent with these findings, Thomson et al. [39] in their hermeneutic phenomenological study reported “person-centered care” as an important intervention approach for adult patients. Similarly, Panes [40] analyzed the concept of family centeredness in the care of patients with SMDs and highlighted mutual participation, professional supportive relationships based on understanding, respect, and empowerment, and individualized care involving family support, which aligns closely with the categories identified in the current study. In another phenomenological study, Skundberg-Kletthagen et al. [41] described the experiences of MHNs using a family-centered care approach, emphasizing the importance of mutual understanding between patients, families, and healthcare providers. These findings are consistent with the second theme in the present study, which suggests placing patients and their families at the heart of psychiatric care for individuals with SMDs. Moreover, Lohrasbi et al. [42] pointed out that in recent years, the responsibility for caring for patients with SMDs has increasingly shifted onto families, underscoring the critical role of family support during hospitalization and beyond. This comparison shows that, despite cultural or systemic differences across healthcare systems, patient- and family-centered care is a globally acknowledged strategy for improving clinical outcomes and fostering better patient cooperation in psychiatric settings.

These findings must be interpreted within the cultural framework of Iran, where family involvement and societal attitudes towards mental illness play a major role in shaping care processes. The Iranian cultural context significantly influences the care process for patients with SMDs, with a strong emphasis on family involvement and collective decision-making. Family support is crucial in facilitating patient cooperation, though it may also present challenges such as conflicting expectations between nurses and families. Additionally, the stigma surrounding mental illness in Iranian society may affect the therapeutic relationship, leading to delays in seeking care and complicating trust-building [43].

The third main theme extracted from the MHNs’ experiences of caring for inpatients with SMDs was “complex and burdensome care,” which encompassed challenges in gender-based care, challenges due to disease symptoms, as well as psychological and physical complications of care. The complexity of care arises from the unpredictability of the disease’s symptoms, which complicate the caregiving process and often lead to psychological strain such as burnout, as well as physical exhaustion. This theme is closely related to job burnout among MHNs due to the intensive care demands. In a qualitative study, Cisneros [44] examined the burnout experiences among psychiatric hospital staff, highlighting excessive workload and additional responsibilities resulting from safety concerns linked to patients’ aggressive behaviors. Similarly, a cross-sectional study conducted in Saudi Arabia by Tununu and Martin [45] reported varying levels of burnout among MHNs, attributing it to the challenges of working with patients exhibiting mental disorders and aggressive behaviors. These findings underline the need for psychological support and effective coping strategies for nurses to maintain their wellbeing and ensure the quality of care for patients.

## Conclusion

The present study was practically significant from three perspectives. First, it highlighted the care of inpatients with SMDs, whose physical and mental health can be severely threatened by the nature of the disease and ineffective care. Furthermore, the patients’ conditions can place a heavy burden on healthcare services and families. Second, the study aimed to explore and explain MHNs’ perceptions and experiences, given their essential role in healthcare services and the promotion of mental health. The third unique aspect of this study was the use of a qualitative approach to investigate the experiences of MHNs in caring for inpatients with SMDs, an area that has been limited in research in Iran.

Based on the findings of the study, which emphasize the importance of care tailored to the nature of the disease, patient- and family-based care, and the complex and burdensome nature of caring for patients with SMDs, it is recommended to organize empowerment courses for MHNs focusing on therapeutic communication skills, crisis management, and medication care. Additionally, family education is crucial to support patients better and build mutual trust and respect. To alleviate the psychological and physical pressures on nurses, hiring support staff and improving the physical structure of psychiatric wards are recommended. Furthermore, monitoring the physical and mental health of nurses and providing salaries that reflect their working conditions can contribute to improving working conditions and care quality. Finally, strategies based on close collaboration with families and attention to individual patient characteristics can enhance treatment outcomes and reduce challenges related to complex care.

One of the limitations of the present study was the small number of male participants, along with the fact that the majority of recruited nurses lacked specialized mental health training. Therefore, it is proposed to conduct further studies on inpatient care for individuals with SMDs using other qualitative approaches, such as phenomenology, grounded theory, and ethnography, to explore additional dimensions. It is also recommended to carry out similar studies involving other healthcare team members and family caregivers of patients with SMDs.

We also acknowledge some methodological limitations. These include the potential for researcher bias influencing data collection and interpretation. Furthermore, content analysis, as a method, may have limitations in fully capturing the complexity of participants’ experiences, as it relies on interpreting text data within predefined categories, possibly overlooking nuances or subtle meanings.

## Electronic supplementary material

Below is the link to the electronic supplementary material.

Supplementary Material 1

## Data Availability

The datasets used and/or analyzed during the current study are available from the corresponding author on reasonable request.

## References

[1] Luciano M, Sampogna G, Amore M, Andriola I, Calcagno P, Carmassi C, Del Vecchio V, Dell’Osso L, Di Lorenzo G, Gelao B. How to improve the physical health of people with severe mental illness? A multicentric randomized controlled trial on the efficacy of a lifestyle group intervention. Eur Psychiatry. 2021;64(1):e72. 10.1192/j.eurpsy.2021.2253. 34812136 10.1192/j.eurpsy.2021.2253 PMC8715281

[2] Sahile Y, Yitayih S, Yeshanew B, Ayelegne D, Mihiretu A. Primary health care nurses attitude towards people with severe mental disorders in addis Ababa, Ethiopia: a cross sectional study. Int J Mental Health Syst. 2019;13(1):1–8. 10.1186/s13033-019-0283-x. 10.1186/s13033-019-0283-x PMC6458759 31011365

[3] Yanos PT, DeLuca JS, Roe D, Lysaker PH. The impact of illness identity on recovery from severe mental illness: A review of the evidence. Psychiatry Res. 2020;288:112950. 10.1016/j.psychres.2020.112950. 32361335 10.1016/j.psychres.2020.112950

[4] Schouten SE, Kip H, Dekkers T, Deenik J, Beerlage-de Jong N, Ludden GD, Kelders SM. Best-practices for co-design processes involving people with severe mental illness for eMental health interventions: a qualitative multi-method approach. Des Health. 2022;6(3):316–44. 10.1080/24735132.2022.2145814.

[5] Schneider F, Erhart M, Hewer W, Loeffler LA, Jacobi F. Mortality and medical comorbidity in the severely mentally ill: a German registry study. Deutsches Ärzteblatt International. 2019;116(23–24):405. 10.3238/arztebl.2019.0405. 31366432 10.3238/arztebl.2019.0405 PMC6683445

[6] Sveinsdottir V, Bond GR. Barriers to employment for people with severe mental illness and criminal justice involvement. J Mental Health. 2020;29(6):692–700. 10.1080/09638237.2017.1417556. 10.1080/09638237.2017.1417556 29265941

[7] Brouwers EP. Social stigma is an underestimated contributing factor to unemployment in people with mental illness or mental health issues: position paper and future directions. BMC Psychol. 2020;8:1–7. 10.1186/s40359-020-00399-0. 32317023 10.1186/s40359-020-00399-0 PMC7171845

[8] Aas CF, Vold JH, Gjestad R, Skurtveit S, Lim AG, Gjerde KV, Løberg E-M, Johansson KA, Fadnes LT. Substance use and symptoms of mental health disorders: a prospective cohort of patients with severe substance use disorders in Norway. Subst Abuse Treat Prev Policy. 2021;16:1–10. 10.1186/s13011-021-00354-1. 33639969 10.1186/s13011-021-00354-1 PMC7912462

[9] Fu X-L, Qian Y, Jin X-H, Yu H-R, Wu H, Du L, Chen H-L, Shi Y-Q. Suicide rates among people with serious mental illness: a systematic review and meta-analysis. Psychol Med. 2023;53(2):351–61. 10.1017/S0033291721001549. 33952359 10.1017/S0033291721001549

[10] El-Monshed A, Amr M. Association between perceived social support and recovery among patients with schizophrenia. Int J Afr Nurs Sci. 2020;13:100236. 10.1016/j.ijans.2020.100236.

[11] Janardhana N, Raghunandan S, Naidu DM, Saraswathi L, Seshan V. Care giving of people with severe mental illness: an Indian experience. Indian J Psychol Med. 2015; 37(2):184–94.10.4103/0253-7176.155619 10.4103/0253-7176.155619 PMC4418252 25969605

[12] Tyerman J, Patovirta A-L, Celestini A. How stigma and discrimination influences nursing care of persons diagnosed with mental illness: A systematic review. Issues Mental Health Nurs. 2021;42(2):153–63.10.1080/01612840.2020.1789788 10.1080/01612840.2020.1789788 32762576

[13] Nossel I, Wall MM, Scodes J, Marino LA, Zilkha S, Bello I, Malinovsky I, Lee R, Radigan M, Smith TE. Results of a coordinated specialty care program for early psychosis and predictors of outcomes. Psychiatric Serv. 2018;69(8):863–70. 10.1176/appi.ps.201700436. 10.1176/appi.ps.201700436 29759055

[14] Uslu E, Buldukoglu K. Randomized controlled trial of the effects of nursing care based on a telephone intervention for medication adherence in schizophrenia. Perspect Psychiatr Care. 2020;56(1). 10.1111/ppc.12376. 10.1111/ppc.12376 30912160

[15] Lundström S, Jormfeldt H, Hedman Ahlström B, Skärsäter I. Mental health nurses’ experience of physical health care and health promotion initiatives for people with severe mental illness. Int J Ment Health Nurs. 2020;29(2):244–53. 10.1111/inm.12669. 31663262 10.1111/inm.12669

[16] Cranage K, Foster K. Mental health nurses’ experience of challenging workplace situations: A qualitative descriptive study. Int J Ment Health Nurs. 2022;31(3):665–76. 10.1111/inm.12986. 35347822 10.1111/inm.12986 PMC9314796

[17] Momen NC, Plana-Ripoll O, Agerbo E, Benros ME, Børglum AD, Christensen MK, Dalsgaard S, Degenhardt L, de Jonge P, Debost J-CP. Association between mental disorders and subsequent medical conditions. N Engl J Med. 2020;382(18):1721–31. 10.1056/NEJMoa1915784. 32348643 10.1056/NEJMoa1915784 PMC7261506

[18] Mehregan M, Khosravi A, Farhadian M, Mohammadi Y. The age and cause decomposition of inequality in life expectancy between Iranian provinces: application of arriaga method. BMC Public Health. 2022;22(1):772. 10.1186/s12889-022-13092-1. 35428278 10.1186/s12889-022-13092-1 PMC9013064

[19] Rastegar H, Morasae EK, Doshmangir L. The dynamics of mental health policy in Iran over the last century. BMC Psychol. 2025;13(1):51. 10.1186/s40359-025-02384-x. 39825394 10.1186/s40359-025-02384-x PMC11742783

[20] Mirghaed MT, Gorji HA, Panahi S. Prevalence of psychiatric disorders in Iran: a systematic review and meta-analysis. Int J Prev Med. 2020;11(1):21. 10.4103/ijpvm.IJPVM_510_18. 32175061 10.4103/ijpvm.IJPVM_510_18 PMC7050223

[21] Noorbala AA, Bagheri Yazdi SA, Faghihzadeh S, Kamali K, Faghihzadeh E, Hajebi A, Akhondzadeh S, Esalatmanesh S, Bagheri Yazdi H, Abbasinejad M, et al. Trends of mental health status in Iranian population aged 15 and above between 1999 and 2015. Arch Iran Med. 2017;20(11 Suppl):S2–6. 29481116

[22] Zarea K, Fereidooni-Moghadam M, Baraz S, Tahery N. Challenges encountered by nurses working in acute psychiatric wards: A qualitative study in Iran. Issues Ment Health Nurs. 2018;39(3):244–50. 10.1080/01612840.2017.1377327. 29064747 10.1080/01612840.2017.1377327

[23] Pazargadi M, Fereidooni Moghadam M, Fallahi Khoshknab M, Alijani Renani H, Molazem Z. The therapeutic relationship in the Shadow: nurses’ experiences of barriers to the Nurse-Patient relationship in the psychiatric ward. Issues Ment Health Nurs. 2015;36(7):551–7. 10.3109/01612840.2015.1014585. 26309175 10.3109/01612840.2015.1014585

[24] Holmberg M, Hammarbäck S, Andersson H. Registered nurses’ experiences of assessing patients with mental illness in emergency care: A qualitative descriptive study. Nordic J Nurs Res. 2020;40(3):151–61. 10.1177/2057158520941753.

[25] Wood L, Williams C, Billings J, Johnson S. The therapeutic needs of psychiatric in-patients with psychosis: A qualitative exploration of patient and staff perspectives. BJPsych Open. 2019;5(3):e45. 10.1192/bjo.2019.33. 31530314 10.1192/bjo.2019.33 PMC6537445

[26] Voort NTvd, Klaessen NC, Poslawsky IE, van Meijel B. Mental health nurses’ perceptions of their role in physical screening and lifestyle coaching for patients with a severe mental illness: a qualitative study. J Am Psychiatr Nurses Assoc. 2024;30(1):141–8. 10.1177/10783903221085596. 35442098 10.1177/10783903221085596

[27] Foronda C. A theory of cultural humility. J Transcult Nurs. 2020;31(1):7–12. 10.1177/1043659619875184. 31516091 10.1177/1043659619875184

[28] Speziale HS, Streubert HJ, Carpenter DR. Qualitative research in nursing: advancing the humanistic imperative. Lippincott Williams & Wilkins; 2011.

[29] Kyngäs H. Inductive Content Analysis. In: The Application of Content Analysis in Nursing Science Research. edn. Edited by Kyngäs H, Mikkonen K, Kääriäinen M. Cham: Springer International Publishing; 2020: 13–21.

[30] Tong A, Sainsbury P, Craig J. Consolidated criteria for reporting qualitative research (COREQ): a 32-item checklist for interviews and focus groups. Int J Qual Health Care. 2007;19(6):349–57. 10.1093/intqhc/mzm042. 17872937 10.1093/intqhc/mzm042

[31] Renz SM, Carrington JM, Badger TA. Two strategies for qualitative content analysis: an intramethod approach to triangulation. Qual Health Res. 2018;28(5):824–31. 10.1177/1049732317753586. 29424274 10.1177/1049732317753586

[32] Graneheim UH, Lindgren B-M, Lundman B. Methodological challenges in qualitative content analysis: A discussion paper. Nurse Educ Today. 2017;56:29–34. 10.1016/j.nedt.2017.06.002. 28651100 10.1016/j.nedt.2017.06.002

[33] Polit D, Beck C. Essentials of nursing research: appraising evidence for nursing practice. Lippincott Williams & Wilkins; 2020.

[34] Lincoln YS, Guba EG. But is it rigorous? Trustworthiness and authenticity in naturalistic evaluation. New Dir Program Evaluation. 1986;1986(30):73–84. 10.1002/ev.1427.

[35] Zarea K, Nikbakht-Nasrabadi A, Abbaszadeh A, Mohammadpour A. Psychiatric nursing as ‘different’care: experience of Iranian mental health nurses in inpatient psychiatric wards. J Psychiatr Ment Health Nurs. 2013;20(2):124–33. 10.1111/j.1365-2850.2012.01891.x. 22384949 10.1111/j.1365-2850.2012.01891.x

[36] Daliri DB, Laari TT, Abagye N, Afaya A. Exploring the experiences of mental health nurses in the management of schizophrenia in the upper East region of Ghana: a qualitative study. BMJ Open. 2024;14(3):e079933. 10.1136/bmjopen-2023-079933. 38503418 10.1136/bmjopen-2023-079933 PMC10952925

[37] Välimäki M, Lantta T, Lam YTJ, Cheung T, Cheng PYI, Ng T, Ip G, Bressington D. Perceptions of patient aggression in psychiatric hospitals: a qualitative study using focus groups with nurses, patients, and informal caregivers. BMC Psychiatry. 2022;22(1):344. 10.1186/s12888-022-03974-4. 35585520 10.1186/s12888-022-03974-4 PMC9118596

[38] Sim IO, Ahn KM, Hwang EJ. Experiences of psychiatric nurses who care for patients with physical and psychological violence: A phenomenological study. Int J Environ Res Public Health. 2020;17(14):5159. 10.3390/ijerph17145159. 32708899 10.3390/ijerph17145159 PMC7400158

[39] Thomson AE, Racher F, Clements K. Person-centered psychiatric nursing interventions in acute care settings. Issues Ment Health Nurs. 2019;40(8):682–9. 10.1080/01612840.2019.1585495. 31074676 10.1080/01612840.2019.1585495

[40] Panes I. Family centeredness in mental health: A concept analysis. J Health Caring Sci. 2020;2(1):48–70. 10.37719/jhcs.2020.v2i1.ra002.

[41] Skundberg-Kletthagen H, Gonzalez MT, Schröder A, Moen ØL. Mental health professionals’ experiences with applying a family-centred care focus in their clinical work. Issues Ment Health Nurs. 2020;41(9):815–23. 10.1080/01612840.2020.1731028. 32401564 10.1080/01612840.2020.1731028

[42] Lohrasbi F, Alavi M, Akbari M, Maghsoudi J. Promoting psychosocial health of family caregivers of patients with chronic mental disorders: A review of challenges and strategies. Chonnam Med J. 2023;59(1):31. 10.4068/cmj.2023.59.1.31. 36794251 10.4068/cmj.2023.59.1.31 PMC9900218

[43] Amin-Esmaeili M, Shadloo B, Rahimi-Movaghar A, Samimi Ardestani SM, Hajebi A, Khatibzadeh S, Sharifi V, Samadi R, Yasamy MT, Zarghami M, et al. Major depressive disorder in Iran: epidemiology, health care provision, utilization, and challenges. Arch Iran Med. 2022;25(5):329–38. 10.34172/aim.2022.54. 35943010 10.34172/aim.2022.54 PMC11904293

[44] Cisneros CJ. Experience of burnout among psychiatric hospital staff. Walden University; 2023.

[45] Tununu AF, Martin P. Prevalence of burnout among nurses working at a psychiatric hospital in the Western cape. Curationis. 2020;43(1):1–7. 10.4102/curationis.v43i1.2117. 10.4102/curationis.v43i1.2117 PMC7479374 32787430

